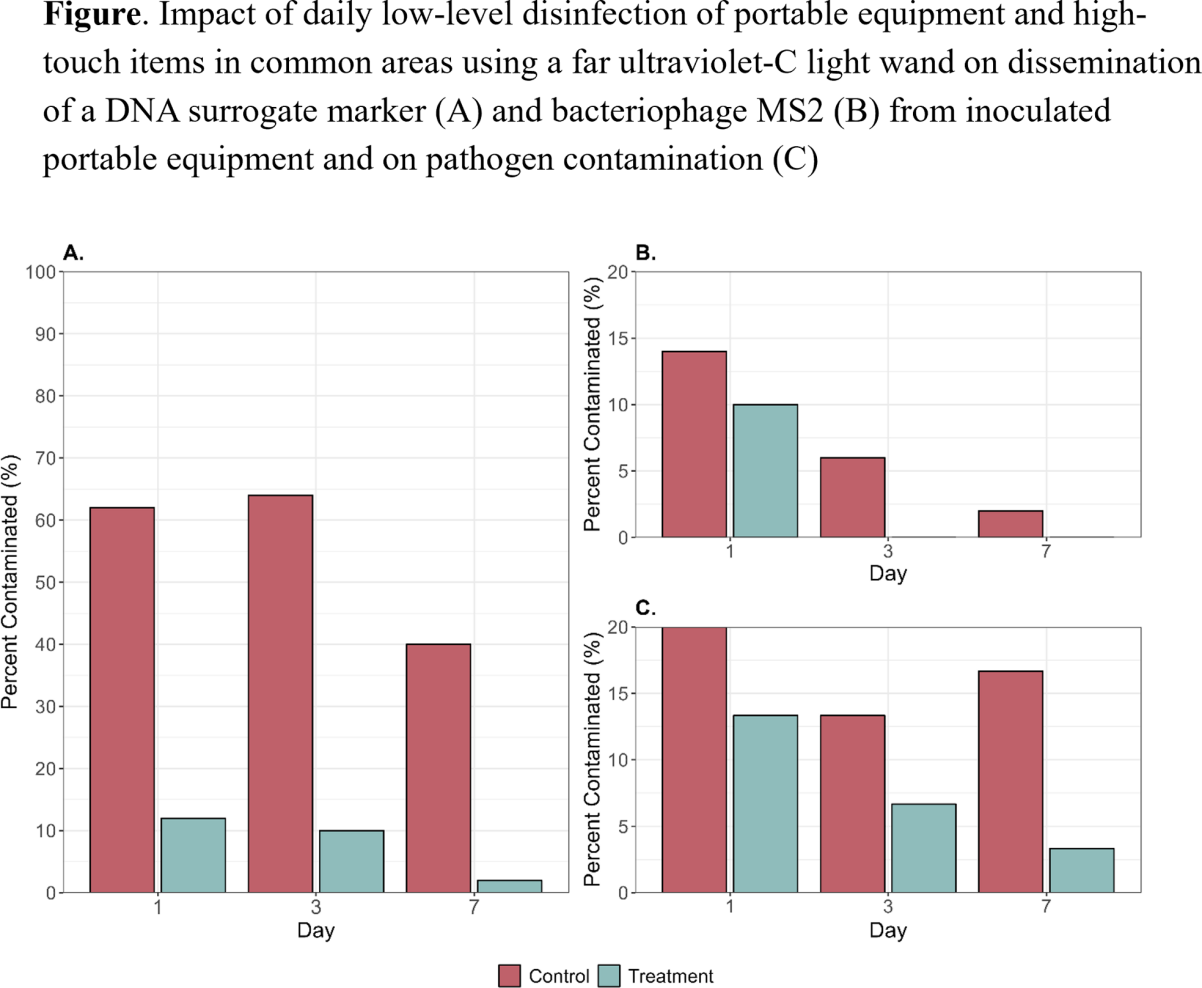# 223 Clinical Characteristics and Genomic Analysis of NDM CRAB Isolates in an Academic Medical Center 2021-2025

**DOI:** 10.1017/ash.2026.10603

**Published:** 2026-06-23

**Authors:** Amelia Milner, Samir Memic, Maria Torres-Teran, Jennifer Cadnum, Curtis Donskey

**Affiliations:** 1 Cleveland VA Medical Center; 2 VA Medical Center

## Abstract

**Background:** Portable medical equipment is a potential source for transmission of healthcare-associated pathogens. However, cleaning and disinfection of equipment is suboptimal in most healthcare facilities. **Methods:** On a long-term care facility (LTCF) ward, we examined the impact of an intervention in which a far ultraviolet-C wand was used to provide low-level disinfection of portable equipment and high-touch items in common areas. The surrogate markers bacteriophage MS2 and cauliflower mosaic virus DNA were inoculated onto 5 portable medical devices in the morning on day 1; in the afternoon on days 1, 3, and 7 swabs for recovery of the markers and culture for pathogens were obtained from 50 surfaces including portable equipment, common areas, and resident rooms. The percentages of sites with contamination were compared for the week of the intervention versus for a control week without far UV-C exposure. **Results:** In the control period, the DNA marker and bacteriophage MS2 disseminated widely and were detected on non-inoculated equipment, the nursing station, and in resident rooms. In comparison to the control period, there was a significant reduction in contamination with the DNA marker during the far UV-C intervention period (P<0.01), and a trend toward reduced contamination with bacteriophage MS2 (P=0.20) and pathogens (P=0.11) (Figure). **Conclusions:** Surrogate markers inoculated onto portable medical equipment disseminated widely throughout a LTCF ward. Daily low-level disinfection using a far UV-C wand reduced dissemination of the surrogate markers and there was a non-significant reduction in pathogen contamination.

**Figure:**